# Gestational week-specific of uterine artery Doppler indices in predicting preeclampsia: a hospital-based retrospective cohort study

**DOI:** 10.1186/s12884-021-04329-9

**Published:** 2021-12-24

**Authors:** Jiang-Nan Wu, Ming-Qing Li, Feng Xie, Bin Zhang

**Affiliations:** 1 Obstetrics and Gynecology Hospital of Fudan University, 566 Fangxie Rd, Shanghai, 200011 China; 2 Shanghai Key Laboratory of Female Reproductive Endocrine-Related Diseases, Shanghai, China

**Keywords:** Uterine artery Doppler, Pulsatility index, Resistance index, Preeclampsia

## Abstract

**Background:**

Plenty of studies explored the relationship between uterine artery (UtA) Doppler indices and the onset of preeclampsia at different trimesters. However, few studies test the gestational week-specific predictive value of the UtA indices for subsequent preeclampsia and compare the difference of right or left UtA indices (e.g., pulsatility or resistance index [PI or RI]).

**Methods:**

Hospital-based retrospective cohort study of singleton pregnant women who received the Doppler test between 2012 and 2016 was conducted in 2018. The predictive performance of the UtA indices for preeclampsia and its variants, including early-onset preeclampsia (< 34 weeks) and preterm preeclampsia (< 37 weeks), was estimated.

**Results:**

The UtA indices, with a cutoff value of 1.11 for the right and left UtA-PI, and 0.66 and 0.63 for the right and left UtA-RI, respectively, were effective predictors for subsequent preeclampsia. The prediction was satisfactory at the 9^th^ week of the Doppler scan: areas under the curve ≥ 0.80, the Youden index ranging from 0.54 to 0.58, the sensitivity of 63.2 ~ 73.7%, and the specificity 84.2 ~ 91.3%, respectively. The UtA indices had comparable performance in screening for early-onset and preterm preeclampsia but showed lower predictive value for late-onset cases. Among these indices, the right UtA-RI had the highest specificity (all P < 0.01), while the left UtA-PI showed good authenticity (higher Youden index) in predicting the disorder.

**Conclusions:**

The second-trimester measured UtA indices had a satisfactory performance at the 9^th^ week in predicting subsequent preeclampsia. The right UtA-RI was the first choice in ruling out preeclampsia, while the left UtA-PI showed the best authenticity of the prediction.

**Supplementary Information:**

The online version contains supplementary material available at 10.1186/s12884-021-04329-9.

## Background

Preeclampsia, a heterogeneous complication affecting 2 to 5% of pregnancies, is a major cause of maternal mortality and morbidity and associated with high risks of fetal adverse outcomes, including perinatal death, preterm delivery, and intrauterine growth restriction [1, 2] The cause of preeclampsia is still unclear, screening for the disorder is therefore important [3, 4]. Failure of effective trophoblast invasion of uterine spiral arteries is linked with the development of preeclampsia and is characterized by an increase of the uterine artery (UtA) indices. Many studies had identified that UtA Doppler indices (e.g., pulsatility index [PI], or resistance index [RI]) show good performance in predicting preeclampsia [5]. However, few studies find an optimal gestational week-specific prediction for the Doppler test in screening for the disorder. Preeclampsia is a heterogeneous complication. The heterogeneity is manifested in different hemodynamics and origins and is associated with the initiation time of the disorder [6]. It is widely accepted to classify the disease into early and late-onset types (e.g., < 34 or ≥ 34 weeks). Early-onset preeclampsia is linked to defective trophoblast invasion with a high percentage of altered uterine artery Doppler [6]. Gestational week-specific prediction of the Doppler test for preeclampsia may help determine an optimal gestation age for the test and might provide evidence for a better understanding of the heterogeneity of the disorder. Furthermore, the UtA indices were classified by the 90^th^/95^th^ percentile of the original data or were standardized in previous studies [3, 7, 8]. Such data processing may result in poor operability and decrease the discrimination of the original data in predicting preeclampsia. Thus, we conducted a hospital-based retrospective cohort study to answer these concerns.

## Methods

### Study design and data collection

We conducted a hospital-based retrospective cohort study of pregnant women who received prenatal examinations at the Obstetrics and Gynecology Hospital of Fudan University between April 2012 and August 2016 in Shanghai, China. Details of the design and data sources have been described elsewhere [9–11]. Briefly, all pregnancies who received prenatal examination at the hospital were followed up till the delivery. Fetal ultrasound examination, including the UtA Doppler, is a routine protocol for pregnant women around 20 to 24 weeks of gestation. Information of maternal characteristics (e.g., maternal age, residence, parity, assisted conception), pregnancy complication, and outcomes (e.g., preeclampsia, birth weight, fetal gender), were collected during the period. Pregnant women who lost to follow-up and who did not take the Doppler test were excluded from the study. Singleton pregnant women were included in the analysis. No missing data (variables or outcomes) was found for women included in the analysis. The study was approved by the Ethics Committee of the Obstetrics and Gynecology Hospital of Fudan University (No. 2017–35, 2017–35-C1).

### Preeclampsia definition

Preeclampsia is defined as the onset of hypertension and proteinuria after 20 weeks of gestation according to the guideline [12]. As in a previous study [13], preeclampsia in the present study included gestational hypertension (hypertension that develops after 20 weeks of gestation without proteinuria), chronic hypertension with superimposed preeclampsia (new onset of proteinuria in women with preexisting hypertension), preeclampsia (bold pressure ≥ 140 mm Hg systolic and/or 90 mm Hg diastolic, and proteinuria ≥ 0.3 g/24 h, or urinary protein/creatinine ratio ≥ 0.3, or random urinary protein ≥ ( +)), and eclampsia (an advanced form of preeclampsia that is characterized by convulsions of unexplained causes) [9, 11, 12]. Early-onset preeclampsia referred to the disorder that occurred < 34 weeks of gestation, while ≥ 34 weeks of gestation for late-onset one.

### UtA Doppler measurement

UtA Doppler, including calculation of the left and right UtA-PI and -RI, was measured by trained sonographers using GE Voluson-E6 or GE Voluson-E8 ultrasound devices (GE Healthcare, Zipf, Austria) according to standardized protocols [14]. The ultrasonographers placed the probe on the lower quadrant of the abdomen after identification of the uterine artery at the intersection with the external iliac artery employing color Doppler. At insonation angles of < 30°, pulsed-wave Doppler was used to obtaining at least three similar flow velocity waveforms for each side. Measurements for both right and left UtA PI/RI were calculated. Intra- and inter-observer repeatability was examined by 95% limits of agreement (Bland–Altman method) [15] using the right UtA-PI data of 50 randomly selected pregnant women without preeclampsia. The result showed that the UtA Doppler measurement had good repeatability (Figure S1).

### Data analysis

UtA-PI and -RI are expressed as the medians (interquartile range, IQR) since the data is non-normal distribution. Categorical data are expressed as n (%) between women with and without preeclampsia. The Mann–Whitney *U*-test and chi-square test or Fisher’s exact test were conducted for continuous and categorical variables between the two groups, respectively. According to the results in a previous meta-analysis, second-trimester measured UtA-RI (> 0.58 or 90^th^ centile) had a sensitivity of 74% and a specificity of 79% in predicting preeclampsia in patients at low risk or unspecified risk [5]. The sample size of 31,291 in the present study, assuming a prevalence of 5% and at a 0.05 significance level, achieved 100% power to detect a change in sensitivity from 0.74 to 0.80 and 98.9% power to detect a change in specificity from 0.79 to 0.80.

Predictive performance of the UtA-PI and -RI for cumulative risk of preeclampsia after the Doppler measurement was assessed by receiver operating characteristic (ROC) curve method, area under the curve (AUC), and corresponding 95% confidence intervals (95%CI) were estimated. Predictive performance was satisfactory when an AUC ≥ 0.80 [16] and more preeclampsia cases were included. The AUCs (95% CI), Youden index, cutoff value and corresponding sensitivity (95% CI), and specificity (95% CI) of the UtA indices at the specific gestational week were estimated when the model was deemed to be the most satisfactory (e.g., AUC ≥ 0.80 and most preeclampsia cases were included).

We separately assess the performance of the UtA indices in predicting early- and late-onset preeclampsia. The association of abnormal perfusion (defined as the UtA-PI or -RI of ≥ the cutoff value, respectively) with early- and late-onset preeclampsia were further estimated by the multinomial logistic regression model, in which potential confounders, such as maternal age at delivery (≤ 24, 25–34, and ≥ 35 years), residence (local or nonlocal), parity (nulliparous or pluriparous), assisted conception (yes or no) and fetal sex (male or female), were included.

The performance of the UtA indices in distinguishing early-onset or preterm preeclampsia cases from normal pregnant women was assessed in the total sample and in subgroups of women who delivered prematurely (e.g., < 37 or < 34 weeks). All statistical tests were conducted using SPSS software (version 22.0, IBM Corp., Armonk, NY, USA). A two-sided P value < 0.01 was considered statistically significant.

## Results

### Baseline characteristics

**Fig. 1:**
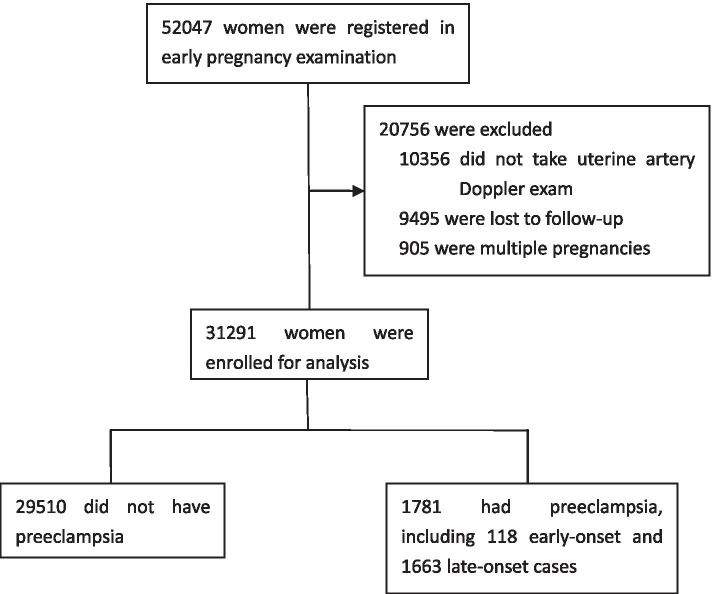
Flow chart of the study

A total of 52,047 pregnant women were registered in their first visit for prenatal examinations at the hospital between April 2012 and August 2016, and 60.1% of them (31,291) who received the Doppler test were included for the analysis (Fig. 1). The majority of women (99.8%, 31,214/31291) received the UtA Doppler at 20 to 24 weeks of gestation, while 23, 37, 10, and 7 women took the test at 19, 25, 26, and 27 weeks of gestation, respectively. The average time for the UtA Doppler test was 22.6 weeks (SD: 0.6). Among these women, 1781 (118) women had developed (early) preeclampsia after the scan, with an incidence rate of 5.7% (0.4%) (Fig. 1). The UtA Doppler indices, including right and left UtA-PI and -RI were significantly higher in whom preeclampsia developed than those in whom it did not. Baseline characteristics except fetal gender were found unbalanced between the groups (Table 1).

**Table 1:** Baseline characteristics of the study participants

Characteristic	No Preeclampsia (*N* = 29,510)	Preeclampsia (*N* = 1781)		
		Early-onset (*N* = 118)	Late-onset (*N* = 1663)	Any (*N* = 1781)
Right PI, median (IQR)	0.79 (0.66–0.96)	1.27 (0.84–1.63) ^a^	0.83 (0.66–1.03) ^a^	0.84 (0.67–1.08) ^a^
Right RI, median (IQR)	0.51 (0.46–0.58)	0.67 (0.53–0.74) ^a^	0.53 (0.46–0.61) ^a^	0.53 (0.46–0.62) ^a^
Left PI, median (IQR)	0.81 (0.67–0.98)	1..27 (0.87–1.73) ^a^	0.86 (0.68–1.09) ^a^	0.87 (0.69–1.12) ^a^
Left RI, median (IQR)	0.52 (0.46–0.59)	0.64 (0.52–0.76) ^a^	0.53 (0.46–0.62) ^a^	0.54 (0.47–0.63) ^a^
Gestational weeks, median (IQR)	39.0 (38.0–40.0)	33.0 (31.0–34.0) ^a^	39.0 (38.0–39.0) ^a^	38.0 (38.0–39.0) ^a^
Local residence no.(%)	23,352 (79.1)	93 (78.8)	1375 (82.7) ^b^	1468 (82.4) ^b^
**Maternal age (year) at delivery year no.(%)**^a^				
< 25	1336 (4.5)	2 (1.7)	59 (3.5)	61 (3.4)
25–34	25,624 (86.8)	94 (79.7)	1374 (82.6)	1468 (82.4)
≥ 35	2549 (8.6)	22 (18.6)	230 (13.8)	252 (14.1)
Nulliparous no. (%)	25,515 (86.5)	96 (81.4)	1504 (90.4) ^a^	1600 (89.8) ^a^
Assisted conception no. (%)	442 (1.5)	6 (5.1) ^b^	52 (3.1) ^a^	58 (3.3) ^a^
Male fetus no. (%)	15,214 (51.6)	56 (47.5)	826 (49.7)	882 (49.5)

^a^*P* < 0.001, ^b^*P* < 0.01 for the difference comparing with no preeclampsia women

### Predictive performance

**Fig. 2:**
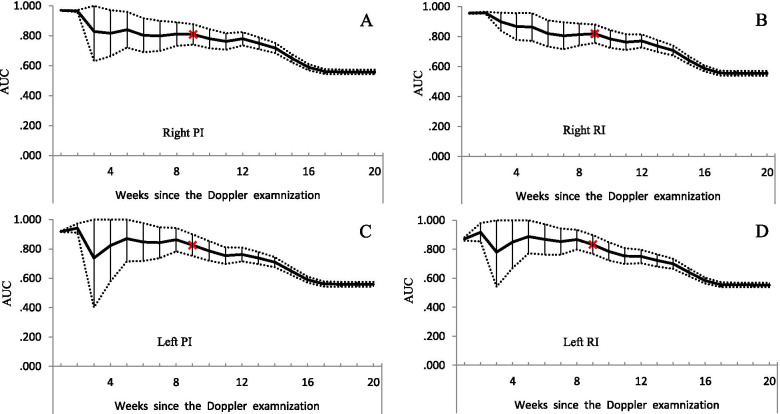
Predictive performance of the uterine artery indices for subsequent preeclampsia. Solid lines represent AUC values, dotted lines were 95% confidence interval of the AUC. Panel **A**, **B**, **C**, and **D** for the predictive performance of right PI, right RI, left PI, and left RI, respectively. PI: pulsatility index; RI resistance index; AUC: area under the curve

**Fig. 3:**
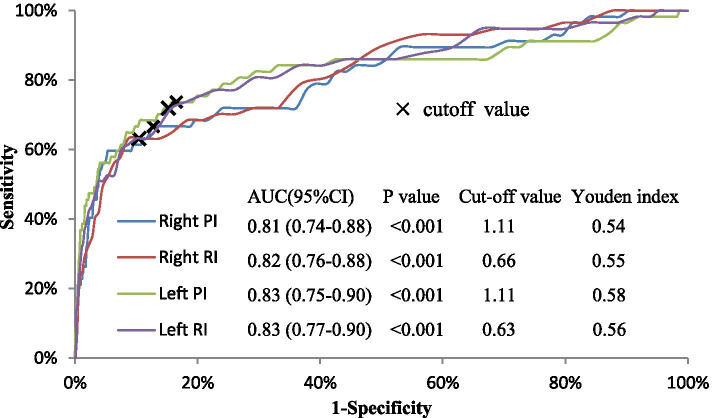
Receiver operating characteristic curve of uterine artery indices in predicting preeclampsia developed within 9 weeks after the Doppler scan. *P* < 0.001 for the difference compared with ^a^ the right PI or ^b^ the right RI. CI: confidence interval; PI: pulsatility index; RI resistance index

Right and left UtA-PI and -RI had similar performance in predicting preeclampsia. Overall, the predictive performance decreased within 3 weeks after the UtA Doppler, and remained stable between the 4^th^ and 9^th^ week of the test (all AUC > 0.80), and continued to decline from the 10^th^ week of the test (Fig. 2). The cutoff values were 1.11 for right and left UtA-PI; and were 0.66 and 0.63 for right and left UtA-RI, respectively (Fig. 3).

The prediction performed best at the 9^th^ week of the scan according to the definition of satisfactory (AUC ≥ 0.80 and most preeclampsia cases were included). A total of 57 cases (3.2% of total cases) were confirmed within 9 weeks after the Doppler scan. The ROC method indicated that the AUCs for right PI and RI, left PI and RI were 0.81, 0.82, 0.83, and 0.83, respectively. The Youden index of right and left UtA-PI were 0.54 and 0.58, and 0.55 and 0.56 for the right and left UtA-RI, respectively (Fig. 3). Sensitivity for the cut-off value of right and left UtA-PI was 66.7% (54.4%-78.9%) and 73.7% (62.3%-85.1%), and corresponding specificity were 87.3% (86.9%-87.6%) and 84.2% (83.8%-84.6%), respectively. Accordingly, sensitivity for the cutoff value of right and left UtA-RI was 63.2% (50.6%-75.7%) and 71.9% (60.3%-83.6%), and the specificity was 91.3% (91.0%-91.6%) and 84.2% (83.8%-84.6%), respectively.

**Fig. 4:**
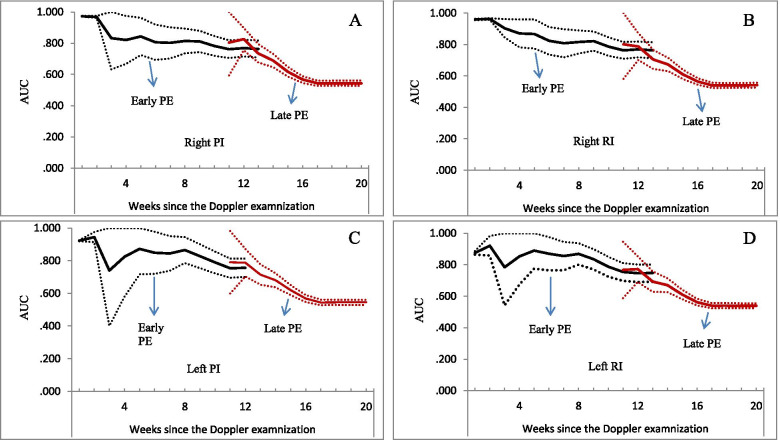
Predictive performance of uterine artery indices for early and late preeclampsia, by weeks after the Doppler scan. Solid lines represent AUC values (black for early PE, red for late PE), dotted lines were 95% confidence interval of the AUC. Panel **A**, **B**, **C**, and **D** for the predictive performance of right PI, right RI, left PI, and left RI, respectively. PI: pulsatility index; RI resistance index; AUC: area under the curve

The predictive performance of the UtA indices for early-onset preeclampsia was better than that for late-onset preeclampsia (Fig. 4). The AUCs for early-onset preeclampsia ranged from 0.747 to 0.973, while 0.539 to 0.826 for late-onset preeclampsia. The relationships of abnormal perfusion with the risk of early-onset preeclampsia were stronger than those for late-onset preeclampsia (Table S1).

### Subgroup analysis

**Fig. 5:**
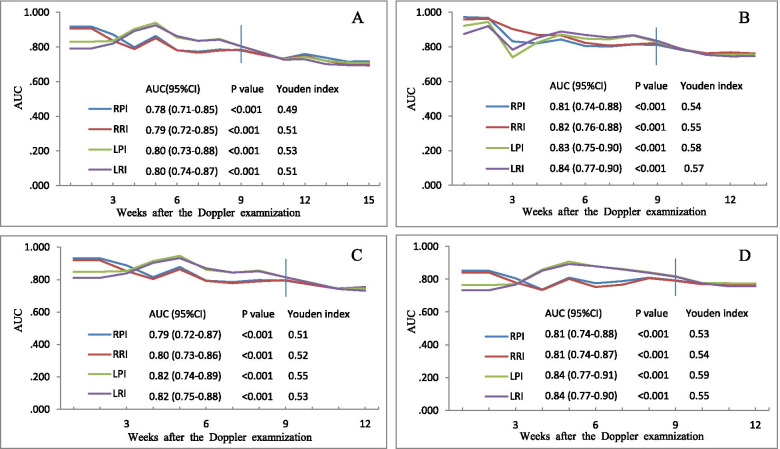
Performance of the uterine artery indices in distinguishing early-onset or preterm preeclampsia from normal pregnant women. The performance (AUC and Youden index) of the cutoff value of the uterine artery indices in predicting early-onset or preterm cases diagnosed within nine weeks after the Doppler scan shown in panel **A** (preterm preeclampsia, gestational week < 37), panel **B** (early-onset preeclampsia, any gestational week), panel **C** (early-onset preeclampsia, gestational week < 37), and panel **D** (early-onset preeclampsia, gestational week < 34). Late-onset preeclampsia cases were excluded from the analysis. RPI and LPI: right and left pulsatility index; RRI and LRI: right and left resistance index; AUC: area under the curve

The 9th-week specific performance of most UtA indices in distinguishing early or preterm preeclampsia from normal pregnant women without preeclampsia remained satisfactory, with most AUCs ≥ 0.80, and the Youden index ranging from 0.49 to 0.59 (Fig. 5). The corresponding sensitivity was 63.2% ~ 80.0% and the specificity was 76.8% ~ 91.6%, 22.9% ~ 64.1% of preeclampsia cases were screened (Table 2). However, the predictive values of the UtA indices for early-onset and preterm preeclampsia cases were not satisfactory (all AUCs < 0.80, Table 3).

**Table 2:** Performance of the uterine artery indices in predicting early-onset or preterm preeclampsia developed within nine weeks after the Doppler scan^*^

Gestational weeks at delivery	No. of women	PE type (No. of total PE cases)	Cases within the 9^th^ week (%)	Uterine artery parameters	Sensitivity (%, 95% CI)	Specificity (%, 95% CI)
Any weeks	29,628	Early PE (118)	57 (48.3%)	Right PI	66.7 (54.4–78.9)	87.7 (87.4–88.1)
				Right RI	63.2 (50.6–75.7)	91.6 (91.3–91.9)^a^
				Left PI	73.7 (62.3–85.1)	84.6 (84.2–85.0)^a,c^
				Left RI	71.9 (60.3–83.6)	84.6 (84.2–85.0)^a,c^
< 37	1484	Preterm PE (236)	54 (22.9%)	Right PI	68.5 (56.1–80.9)	79.9 (77.9–82.0)
				Right RI	64.8 (52.1–77.6)^a^	85.9 (84.1–87.1)^a^
				Left PI	75.9 (64.5–87.3)^d^	77.3 (75.1–79.4)^b,c^
				Left RI	74.1 (62.4–85.8)^d^	77.3 (75.2–79.5)^b,c^
< 37	1354	Early PE (106)	54 (50.9%)	Right PI	68.5 (56.1–80.9)	82.2 (80.1–84.2)
				Right RI	64.8 (52.1–77.6)	87.5 (85.7–89.3)^a^
				Left PI	75.9 (64.5–87.3)	79.3 (77.1–81.5)^a,c^
				Left RI	74.1 (62.4–85.8)	79.3 (77.1–81.5)^a,c^
< 34	274	Early PE (78)	50 (64.1%)	Right PI	72.0 (60.0–84.4)	80.8 (75.6–86.0)
				Right RI	68.0 (55.1–80.9)	86.2 (81.6–90.7)^d^
				Left PI	80.0 (68.9–91.1)	78.6 (73.2–83.9)^c^
				Left RI	78.0 (66.5–89.5)	76.8 (71.3–82.3)^c^

^*^Late-onset cases were excluded from the models

^a^*P* < 0.001, ^b^*P* < 0.01, ^d^*P* < 0.05 for the difference comparing with the RPI; ^c^*P* < 0.001 comparing with the RRI

**Table 3:** Performance of the uterine artery indices in distinguishing early-onset or preterm preeclampsia from variants of normal pregnant women without preeclampsia^*^

Gestational weeks at delivery	No. of women	PE type (cases)	Uterine artery parameters	AUC (95%CI)	*P* value	Youden index
Any weeks	29,628	Early PE (118)	Right PI	0.76 (0.71–0.82)	< 0.001	0.45
			Right RI	0.76 (0.71–0.81)	< 0.001	0.44
			Left PI	0.76 (0.70–0.81)	< 0.001	0.43
			Left RI	0.75 (0.69–0.80)	< 0.001	0.43
< 37	1484	Preterm PE (236)	Right PI	0.72 (0.68–0.76)	< 0.001	0.34
			Right RI	0.70 (0.66–0.74)	< 0.001	0.30
			Left PI	0.71 (0.67–0.75)	< 0.001	0.32
			Left RI	0.69 (0.65–0.73)	< 0.001	0.31
< 37	1354	Early PE (106)	Right PI	0.75 (0.70–0.81)	< 0.001	0.44
			Right RI	0.75 (0.70–0.81)	< 0.001	0.44
			Left PI	0.75 (0.69–0.80)	< 0.001	0.43
			Left RI	0.73 (0.67–0.79)	< 0.001	0.41
< 34	274	Early PE (78)	Right PI	0.77 (0.70–0.84)	< 0.001	0.48
			Right RI	0.77 (0.70–0.83)	< 0.001	0.46
			Left PI	0.78 (0.71–0.84)	< 0.001	0.48
			Left RI	0.76 (0.69–0.83)	< 0.001	0.43

^*^The cutoff values for right PI/RI, left PI/RI are 1.11, 0.66, 1.11, and 0.63, respectively

## Discussion

In this hospital-based retrospective cohort study, we assessed the gestational week-specific predicting performance of the UtA indices for preeclampsia. We identified that the UtA indices in isolation, with a cutoff value of 1.11 for the right and left UtA-PI, and 0.66 and 0.63 for the right and left UtA-RI, respectively, performed satisfactorily at the 9^th^ week after the Doppler test in screening for subsequent preeclampsia, as well as for early-onset and preterm preeclampsia. Furthermore, left UtA-PI had the highest authenticity of the 9th-week specific prediction for preeclampsia (higher Youden index), while right UtA-RI performed better in ruling out the disorder (higher specificity).

Different from the purpose of the first-trimester UtA Doppler in identifying pregnant women for potential preventative treatment (e.g., aspirin), the second-trimester UtA Doppler test focuses on finding target women for intensive monitoring of the pregnancies [17, 18]. The UtA Doppler indices measured at the second trimester are effective markers in screening for preeclampsia, especially for a serious variant of the disease (e.g., early-onset or preterm cases) [3, 5, 8]. However, little is known about the gestational week-specific value of this prediction. The findings of the present study responded to this concern and identified the gestational age characteristics of this prediction.

Because women received the Doppler test at an average of 22.6 weeks of gestation, the UtA indices had a satisfactory performance at the 9^th^ week after the test equivalent to at 31.6 weeks of gestation. Therefore, 24–25 weeks of gestation might be an appropriate time for the Doppler test to screen for all early-onset cases when taking the threshold for the definition (e.g., < 34 weeks) into account. Accordingly, 27–28 weeks of gestation might be an optimal choice for the indices in predicting preterm preeclampsia. In addition, we found that the second-trimester measured UtA indices had better performance in predicting early- than late-onset preeclampsia. This prediction disparity may be largely attributed to the heterogeneity of the disorder [6]. However, the UtA indices had comparable performance in screening for early-onset preeclampsia and preterm cases. These findings aroused our concern about the rationality of the threshold for the definition of early-onset preeclampsia. This was consistent with the results of our previous study, in which we found that a criterion of < 36 weeks of gestation might be a better threshold for the definition of early-onset preeclampsia than the < 34 weeks criterion. Further studies on the threshold for the definition of early-onset preeclampsia were warranted.

In a previous meta-analysis, the performance of the UtA indices (e.g., RI, PI, notching in isolation or in combination) in predicting preeclampsia was pooled and compared. They concluded that a PI is the most predictive Doppler index [5]. The present study further compared the prediction across right and left UtA-PI or -RI. We found some interesting information that might be covered up if the mean of right and left UtA PI/RI values was taken for analysis. The left UtA indices had higher sensitivity in screening for the disease, and the left UtA-PI had the highest authenticity of the prediction. On the contrary, the right UtA Doppler indices performed better in ruling out the disorder. The right UtA-RI had a significantly higher specificity than the other Doppler indices. The reasons for these predictive disparities among the UtA indices are still unknown, might be related to the impact of the placental site on flow impedance [19, 20]. However, these findings may help clinicians optimize the selection of the UtA indices for preeclampsia screening based on different purposes. Furthermore, specific cutoff values of the Doppler indices might be easier to remember and more operational for clinicians, and then improve clinical applicability.

In this cohort study of a large sample of singleton pregnancies, we identified a turning time point of the prediction of preeclampsia for the UtA Doppler indices and compared the performance of the four indices in predicting the disorder. At the same time, our study has some limitations. First, this was a single-center study of singleton pregnancies and only those who received the UtA Doppler (75.2% of women who had been followed up and 60.1% of the total sample) were included in the analysis. The representativeness of the sample might limit the generalizability of the findings. However, we think the results are robust since the sample remained large, the UtA Doppler measurement had good repeatability, and no difference in the incidence of preeclampsia was found between the included and excluded sample (*P* = 0.92). Second, we cannot rule out the impact of the use of aspirin on the results although preventative treatment of aspirin for preeclampsia for pregnancies has no significant effect in the second trimester [21, 22]. In such cases, the performance of the UtA indices in predicting early preeclampsia may underestimate since aspirin has been shown an effective intervention on the risks of early preeclampsia [22]. Finally, the Doppler indices may show different performances between high and low-risk women [5]. But no related stratification analysis was done since we did not obtain maternal factors (e.g., maternal history of preeclampsia) that could be used to classify women into high or low risk.

In conclusion, the second-trimester measured UtA indices performed satisfactorily at the 9^th^ week in predicting subsequent preeclampsia. The left UtA-PI, with a cutoff value of 1.11, had the highest authenticity of the prediction, while the right UtA-RI, with a cutoff value of 0.66, was the first choice in ruling out the disorder.

## Supplementary Information

**Additional file 1:** **Figure S1.** Intra-observer and inter-observer repeatability of the right uterine artery pulsatility index measurement. **Table S1. **Adjusted odds ratio of early and late onset preeclampsia ^a^.

## Data Availability

The datasets used in the present study are available from the corresponding author (wjnhmm@126.com) on reasonable request only.

## Abbreviations

AUC: Area under curve
CI: Confidence interval
NPV: Negative predictive value
PPV: Positive predictive value
PE: Preeclampsia
PI: Pulsatility index
RI: Resistance index
ROC: Receiving operating characteristic
UtA: Uterine artery
