# Supplementary material for: Gestational week-specific of uterine artery Doppler indices in predicting preeclampsia: a hospital-based retrospective cohort study
Source: BMC Pregnancy Childbirth. 2021 Dec 24;21:843. doi: 10.1186/s12884-021-04329-9 (PMC8705461; doi:10.1186/s12884-021-04329-9)
Supplement: Supplementary file 1 — Additional file 1: Figure S1. Intra-observer and inter-observer repeatability of the right uterine artery pulsatility index measurement. Table S1. Adjusted odds ratio of early and late onset preeclampsia a. [file 12884_2021_4329_MOESM1_ESM.docx]

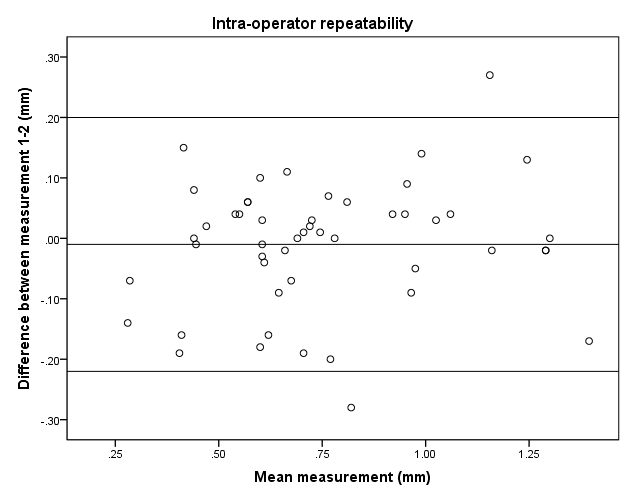

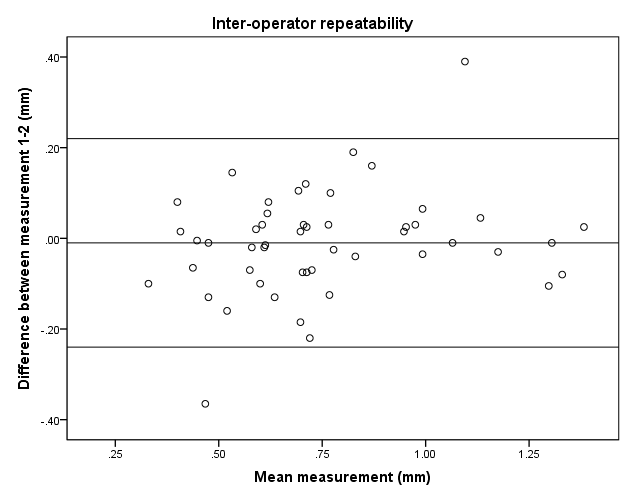


Figure S1. Intra-observer and inter-observer repeatability of the right uterine artery pulsatility index measurement.

Table S1 Adjusted odds ratio of early and late onset preeclampsia ^a^

| Parameters | | Early preeclampsia | Late preeclampsia |
| --- | --- | --- | --- |
|  |  | Adjusted odds ratio (95% CI) | Adjusted odds ratio (95% CI) |
| Right PI | <1.11 | 1.00 | 1.00 |
|  | ≥1.11 | 9.90 (6.85-14.31) | 1.95 (1.72-2.20)^b^ |
| Right RI | <0.66 | 1.00 | 1.00 |
|  | ≥0.66 | 12.11 (8.41-17.44) | 1.85 (1.60-2.14)^b^ |
| Left PI | <1.11 | 1.00 | 1.00 |
|  | ≥1.11 | 7.89 (5.46-11.41) | 1.70 (1.51-1.92)^b^ |
| Left RI | <0.63 | 1.00 | 1.00 |
|  | ≥0.63 | 7.78 (5.38-11.25) | 1.64 (1.45-1.84)^b^ |

^a^ Multinomial logistic regression was conducted, in which maternal age at delivery (<25, 25-34, or ≥ 35), residence (local or nonlocal), parity (nulliparous or pluriparous), assisted conception (yes or no) and fetal sex (male or female) were adjusted.

^b^ P<0.001 compared with the adjusted odds ratio for early preeclampsia.
